# Tripchlorolide induces autophagy in lung cancer cells by inhibiting the PI3K/AKT/mTOR pathway and improves cisplatin sensitivity in A549/DDP cells

**DOI:** 10.18632/oncotarget.19201

**Published:** 2017-07-12

**Authors:** Li-Min Chen, Tian-Jiao Song, Jian-Hong Xiao, Zheng-Hui Huang, Yong Li, Ting-Yan Lin

**Affiliations:** ^1^ Department of Respiratory Medicine, Fujian Medical University Union Hospital, Fuzhou, Fujian 350001, People's Republic of China; ^2^ Department of Geriatrics, Fujian Medical University Union Hospital, Fuzhou, Fujian 350001, People's Republic of China; ^3^ Fujian Institute of Geriatrics, Fujian Medical University Union Hospital, Fuzhou, Fujian 350001, People's Republic of China; ^4^ Department of Respiratory Medicine, Mindong Hospital of Ningde City, Fu'an, Fujian 355000, People's Republic of China

**Keywords:** tripchlorolide, autophagy, lung cancer, PI3K/AKT/mTOR, MDR1

## Abstract

Tripchlorolide (T4) has been shown to induce A549 lung cancer cell death predominantly by activating an autophagy pathway. However, the underlying mechanism remains unclear. Herein, we demonstrated that compared with T4 treatment alone, pretreatment with wortmannin (an inhibitor of phosphatidylinositol 3-kinase), perifosine (an inhibitor of AKT) or rapamycin (an inhibitor of mTOR) combined with a subsequent T4 treatment significantly impaired the cell viability of A549 and A549/DDP lung cancer cells. We found that either treatment scheme markedly reduced the activity of P13K and AKT. Expression of LC3II increased in parallel to the increase of the T4 concentration in both A549 and A549/DDP cells and was repressed by overexpression of AKT. The expression levels of PI3-K, PI3-P, AKT, TSC2, mTOR, p70S6K and 4E-BP1 were minimally affected by the wortmannin, perifosine, or rapamycin plus T4 treatments, but their phosphorylated products were greatly affected in A549 lung cancer cells and slightly affected in A549/DDP lung cancer cells. These results indicate that T4 induces autophagy in lung cancer cells by inhibiting the PI3K/AKT/mTOR signaling pathway. We further found that T4 decreased expression of MDR1 and improved cisplatin sensitivity of A549/DDP cells. Altogether, these results have meaningful implications for tumor therapy in the future.

## INTRODUCTION

Lung cancer is a highly prevalent disease that has been ranked among the leading causes of death worldwide. The incidence of lung cancer has increased by 465% during the past three decades, and the five-year survival rate is less than 15% [1]. Therefore, the medical community has prescribed lung cancer patients chemotherapy, which consists of a single third-generation drug and a platinum drug, to improve survival, disease control, and quality of life [2]. However, due to the resistance to chemotherapeutic drugs, the effectiveness of chemotherapy in the treatment of lung cancer has been unsatisfactory, which is mainly attributed to the dysregulation of death signals in cells [3]. Thus, cancer patients were treated with higher doses of toxic anticancer drugs, which resulted in adverse side effects, and drug resistance reversal has become one of the most attractive approaches for improving the therapeutic efficacy in NSCLC patients [4]. An additional fundamental adverse effect is that these toxic anticancer drugs affect the apoptotic mechanisms in cancer cells, rendering them insensitive to apoptosis, which is a major mode of cell death [5]. Therefore, it is imperative to identify alternative methods of killing tumor cells.

Recently, autophagy, which is a new programmed cell death or type II cell death, has attracted increasing attention due to its important regulatory role in cell death [6]. Autophagy involves a salvage pathway that damages organelles and delivers them to lysosomes for energy and nutrient recycling [7]. Autophagy is characterized by the appearance of double- or multiple-membrane cytoplasmic vesicles that engulf bulk cytoplasm and/or cytoplasmic organelles, such as mitochondria and endoplasmic reticulum. In the initial stage of autophagy, intracellular biological macromolecules are wrapped and engulfed by double-membrane-bound structures called autophagosomes (early autophagic vesicles), which are then transported to the lysosomes to form autolysosomes (late autophagic vesicles) by fusing with the lysosomes [8–10]. The biological macromolecules are then degraded in the autolysosomes and recycled to sustain cellular metabolism [11].

Several studies have documented that autophagy is associated with a variety of diseases, including cancer [12–15], and can be observed in cancer cells upon treatment with chemotherapeutic agents, such as cisplatin, paclitaxel and other cytotoxic drugs [16–18]. More recent studies have reported a close correlation between cancer and reduced autophagic activities. Canuto and Kisen found that cells from chemical carcinogen-induced primary hepatocellular carcinomas or preneoplastic liver nodules had a decreased autophagic capacity compared with normal liver cells [19]. Ahlberg and Yucel reported that autophagic vesicles isolated from liver nodules and hepatocellular carcinomas showed lower activities of lysosomal enzymes than those isolated from normal liver cells [20, 21]. Therefore, efficient, non-toxic and target-specific agents exploiting this cell autophagic pathway and its underlying mechanism have potential applications in the treatment of cancer.

Medicinal plants are a rich source of bioactive compounds for therapeutic agents and currently provide ingredients for 75% of prescribed drugs worldwide [22]. For example, natural extracts from tripterygium have strong immunosuppressive and anti-inflammatory activities and are widely used in the treatment of autoimmune-related diseases. One of these extracts, triptolide, has been reported to induce apoptosis in multiple myeloma cells by inhibiting NF-κB activity [23] and the proliferation of lung cancer cells [24]. However, its clinical application is limited due to its toxic effects. To overcome this deficiency, tripchlorolide (T4), which is an attenuated monomer, has been extracted from tripterygium or obtained from the hydroxyl acylation and chlorination of triptolide and presents higher activity and lower toxicity than triptolide.

In our previous study, we showed that 200 nM of tripchlorolide induced the death of A549 lung cancer cells predominantly through the activation of an autophagy pathway instead of an apoptosis pathway [25]. In this study, we investigated whether tripchlorolide regulated autophagy in A549 lung cancer cells and A549/DDP cisplatin-resistant cell lines through the PI3K/AKT/mTOR signaling pathway to provide a theoretical basis for the application of tripchlorolide in the treatment of cisplatin-resistant lung cancer.

## RESULTS

### Tripchlorolide induces cell death in both A549 and A549/DDP cells primarily by autophagy

**Figure 1:**
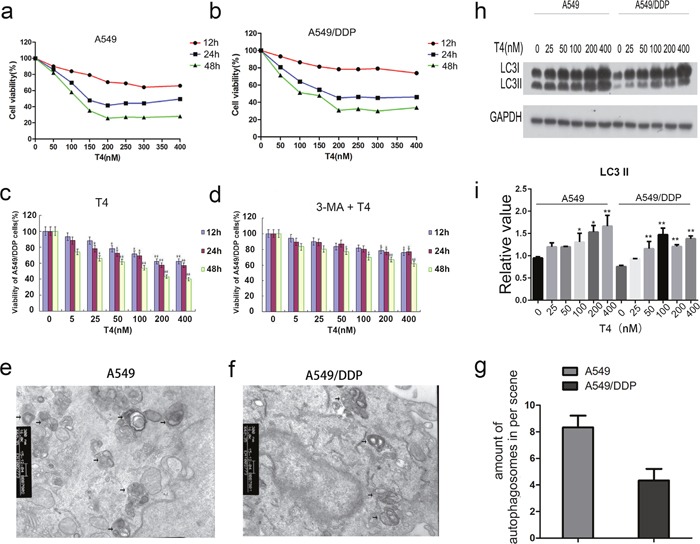
T4-accelerated cell death of A549 and A549/DDP cells and decreased T4 toxicity by 3-MA+T4 and T4-induced cell autophagy in A549 and A549/DDP cells by TEM and western blotting **(a** and **b)** The viability of A549 and A549/DDP cells was reduced with increasing T4 concentrations and incubation times. **(c** and **d)** WhenA549/DDP cells were pretreated with 3-MA, the T4 toxicity was also dramatically reduced (*: P<0.05, **: P<0.01). **(e, f** and **g)** Using TEM, more autophagosomes were observed in A549 cells than in A549/DDP cells after the T4 treatment (Black arrow represents autophagosomes). **(h** and **i)** Using western blotting, T4 was observed to increase LC3II expression in A549 and A549/DDP cells (*: P<0.05, **: P<0.01).

The T4 treatment promoted the death of A549 and A549/DDP cells. A549 and A549/DDP cells were treated with a series of concentrations of T4 (0-400 nM) for different periods (12-48 h). The viability of A549 and A549/DDP cells was equally reduced with increasing T4 concentrations and incubation times (Figure 1a and 1b). After exposure to 200 nM of T4 for 24 h, the cell viability declined to approximately 50%. These results suggest that T4 induces the death of A549 and A549/DDP cells in a dose- and time-dependent manner. In our previous study, we found that when A549 cells were pretreated with 3-MA, which is an inhibitor of autophagy, the T4 toxicity was dramatically reduced. Therefore, in the current study, we observed the dramatic changes in T4 toxicity when A549/DDP cells were pretreated with 3-MA (Figure 1c and 1d). Compared with the controls, the cells that received the 3-MA pretreatment plus the subsequent T4 treatment revealed no significant differences in the cell viability, except for those exposed to 200 nM and 400 nM of T4 for 24-48 h. This finding indicates that when autophagy is inhibited, T4 produces mild toxic effects on the growth of A549/DDP cells.

According to the results of our previous study [25], the IC50 of A549 and A549/DDP cells is 200 nM after 24 h; therefore, we chose 200 nM for 24 h as the reaction concentration for the subsequent experiments. In these experiments, the formation of autophagosomes was examined in T4-treated cells using TEM (Figure 1e, 1f and 1g). In these cells, damaged organelles were observed, such as swollen mitochondria surrounded by double-membrane vacuoles, which subsequently formed autophagosomes. The autophagosomes subsequently fused with a lysosome, and the internal material was degraded. Undegraded debris within the autolysosomes was also observed. Notably, more autophagosomes were present in A549 cells than in A549/DDP cells. The observed results were further verified at the protein level. LC3 is the marker of choice for detecting double-membraned autophagosomes in yeast and mammalian cells. The phosphatidylethanolamine-conjugated form of LC3II is localized in the inner and outer membranes of the phagophore. During the maturation of the autophagosome, LC3 on the outer membrane is cleaved and recycled, whereas LC3 is bound to the inner membrane and remains *in situ* as the autophagosome fuses with the lysosome [26]. Western blotting analysis revealed that the protein level of LC3II was upregulated as the concentration of T4 increased in A549 cells and A549/DDP cells (Figure 1h and 1i). This finding is consistent with the TEM results, which show more LC3II in A549 cells than in A549/DDP cells. Altogether, the above-mentioned findings indicate that T4 induces cell death in A549 and A549/DDP cells, primarily by autophagy.

### Tripchlorolide induces autophagy mainly via the PI3K/AKT/mTOR signaling pathway

**Figure 2:**
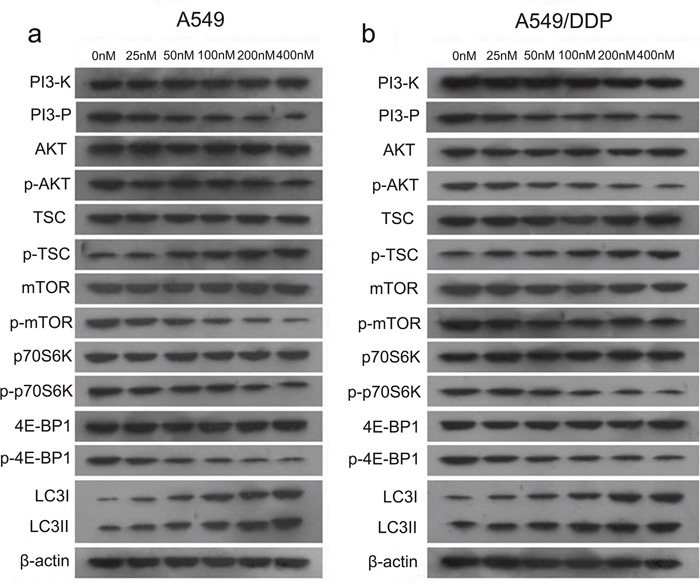
Changes in the PI3K/AKT/mTOR signaling pathway with increasing doses of T4 in A549 and A549/DDP cells **(a** and **b)** The expression levels of PI3-K, PI3-P, AKT, TSC2, mTOR, p70S6K and 4E-BP1 remained almost unchanged, but their phosphorylation levels changed significantly. The expression levels of LC3I and LC3II were also changed.

**Figure 3:**
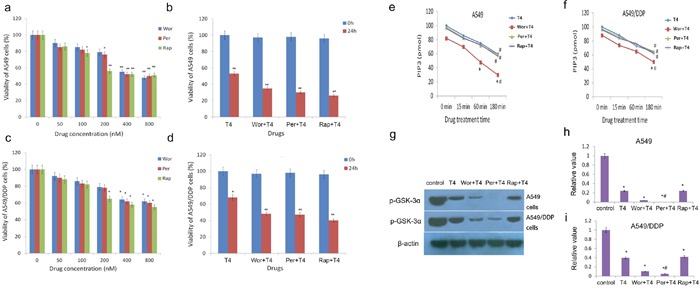
Decreased viability of A549 and A549/DDP cells following the Rap+T4, Wor+T4, and Per+T4 treatments and the altered activity of PI3K and AKT following the T4, Rap+T4, Wor+T4, and Per+T4 treatments **(a** and **c)** The pretreatment concentrations of rapamycin, wortmannin and perifosine were 200, 400 and 400 nM, respectively (*: P<0.05, **: P<0.01). **(b** and **d)** T4 produced a better autophagic effect on the lung cancer cells when combined with a pretreatment of rapamycin, wortmannin and perifosine (*: P<0.05, **: P<0.01). **(e** and **f)** The activity of PI3K was sensitive to the T4, Rap+T4, Wor+T4, and Per+T4 treatments in a time-dependent manner (#: compared with control (0 min), *: P<0.05.). **(g, h** and **i)** The effects of T4, wortmannin, perifosine and rapamycin on the expression of p-GSK-3α (#: compared with T4, *: P<0.05).

According to the molecular literature, the PI3K-AKT signaling pathway is the primary pathway for the survival of cancerous cells and is highly activated in a variety of tumor tissues; the PI3K/AKT/mTOR signaling pathway is the main regulatory pathway that negatively regulates autophagy. In a western blotting experiment, we measured the protein expression levels of PI3-K, PI3-P, AKT, TSC2, mTOR, p70S6K, 4E-BP1, and LC3 in A549 cells and A549/DDP cells after the T4 treatment. The results showed that as the drug concentration increased, there were no differences in the protein levels of PI3-K, PI3-P, AKT, TSC2, mTOR, p70S6K and 4E-BP1, but their phosphorylation levels decreased(except for p-TSC2), and the level of LC3II and p-TSC2increased (Figure 2a and 2b). These results suggest that tripchlorolide may induce autophagy in A549 and A549/DDP lung cancer cells through the inhibition of the PI3K/AKT/mTOR signaling pathway. We further verified the involvement of this targeted signaling pathway in the autophagy by employing inhibitors of PI3K, AKT and mTOR, i.e., wortmannin, perifosine and rapamycin, respectively. The effects of these inhibitors were assayed on cell growth inhibition to confirm the optimal concentrations. The results of the CCK8 assay showed that the viability was reduced with increasing concentrations of the inhibitors and that the viability was reduced significantly at the concentrations of 200, 400 and 400 nM of rapamycin, wortmannin and perifosine, respectively. However, the viability was not significantly reduced when the concentrations of the inhibitors exceeded 400 nM. Therefore, the pretreatment concentrations of rapamycin, wortmannin and perifosine were 200, 400 and 400 nM, respectively, in the follow-up experiments (Figure 3a and 3c). The viability was significantly reduced when the cells were treated with T4 at a concentration of 200 nM for 24 h, but it was more drastically reduced when the cells were pretreated with rapamycin, wortmannin or perifosine for 1 h, followed by the T4 treatment, suggesting that T4 has a better effect on the viability of A549 and A549/DDP lung cancer cells when combined with a pretreatment with rapamycin, wortmannin and perifosine (Figure 3b and 3d). Interestingly, the viability of A549 lung cancer cells was more dramatically reduced than that of A549/DDP lung cancer cells when both cell lines were treated with the same concentrations of the drugs. Due to the main role of the PI3K/AKT/mTOR signaling pathway in autophagy, the activity of PI3K is important for the initiation of autophagy. We examined the effects of T4 and inhibitors of the PI3K/AKT/mTOR signaling pathway on the activity of PI3K to indirectly evaluate the role of PI3K activity in autophagy by quantifying PIP3 (Figure 3e and 3f). The results showed that the activity of PI3K was sensitive in a time-dependent manner to both the T4 treatment and the pretreatment with wortmannin, perifosine, or rapamycin plus the subsequent T4 treatment. PIP3 was most sensitive to the pretreatment with wortmannin plus the subsequent T4 treatment. No significant differences were observed among the T4, perifosine plus T4 and rapamycin plus T4 treatments. The activity of PI3K significantly decreased in A549 and A549/DDP lung cancer cells and, to a lesser extent, A549/DDP cells. Meanwhile, we measured the activity of AKT, which was determined in a kinase reaction using recombinant GSK-3α as the substrate. The phosphorylation of GSK-3α was analyzed by western blotting, and the activity of AKT was indirectly assayed by detecting the expression of p-GSK-3α. The results showed that the activity of AKT was positively correlated with the level of p-GSK-3α and that the activity of AKT in A549 and A549/DDP lung cancer cells was significantly reduced when the cells received both treatment schemes. The activity was the lowest when the cells were pretreated with perifosine, followed by the T4 treatment, and almost no effect on the activity of AKT was observed following the rapamycin pretreatment plus the subsequent T4 treatment. The effects of T4, wortmannin, perifosine and rapamycin on the expression of p-GSK-3α showed the same trend in both A549 and A549/DDP lung cancer cells (Figure 3g and 3h and 3i).

**Figure 4:**
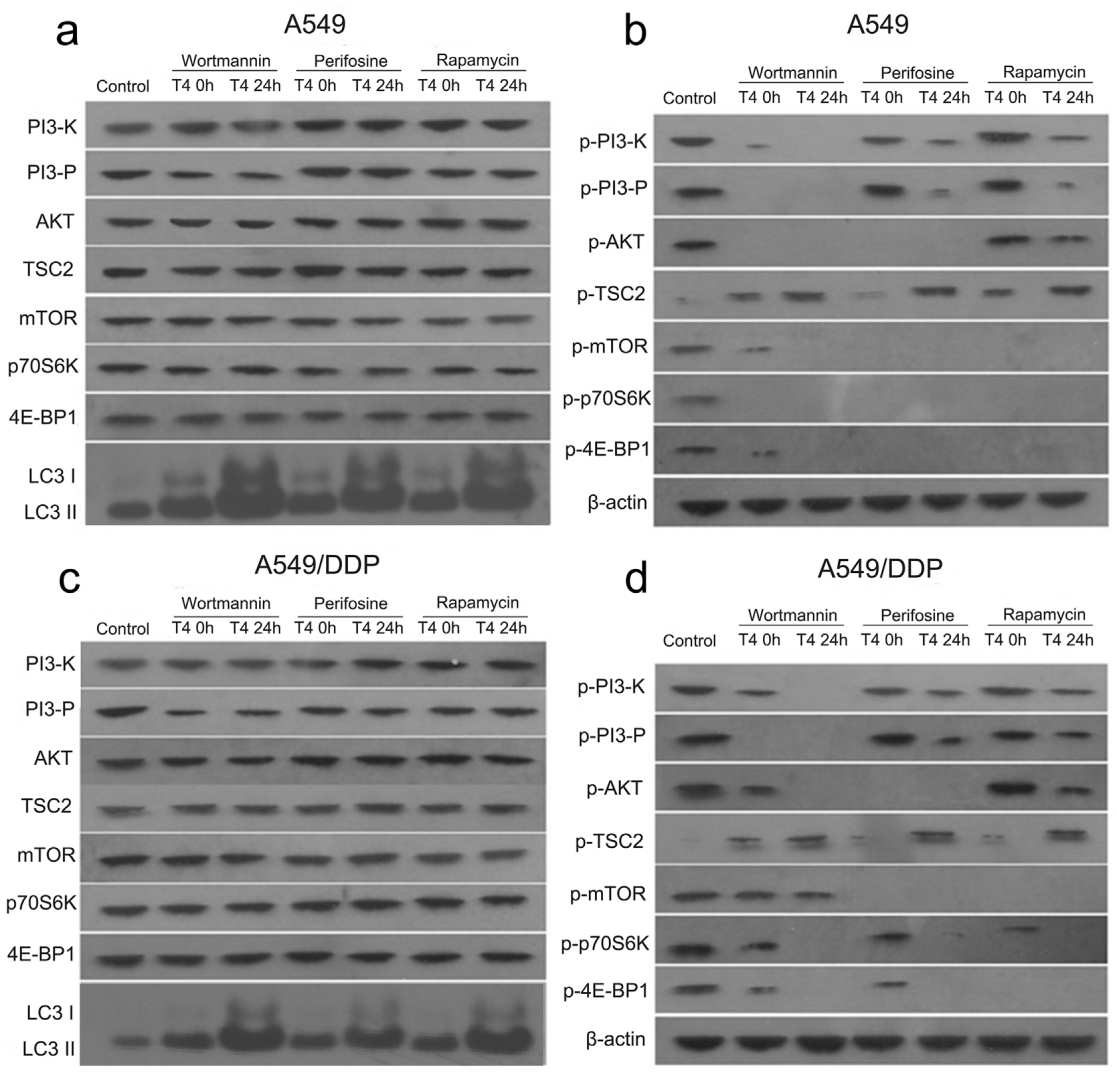
Changes in the PI3K/AKT/mTOR signaling pathway in A549 and A549/DDP cells following the Rap+T4 (0 h, 24 h), Wor+T4 (0 h, 24 h), and Per+T4 (0 h, 24 h) treatments **(a)** Following the Rap+T4 (0 h, 24 h), Wor+T4 (0 h, 24 h), and Per+T4 (0 h, 24 h) treatments, no differences were evident in the expression of PI3-K, PI3-P, AKT, TSC2, mTOR, p70S6K and 4E-BP1 in A549 lung cancer cells. No expression of LC3 in the control was observed; however, the abundant expression of LC3 was detected following the Rap+T4 (24 h), Wor+T4 (24 h), and Per+T4 (24 h) treatments. **(b)** Very low expression levels of phosphorylated PI3-K (p-PI3-K) were observed following the Rap+T4, Wor+T4, and Per+T4 treatments. No expression of p-PI3-K was observed in the Wor+T4 group at 24 h. Regarding p-AKT, no significant differences were found between the Rap+T4 group and the control group, and no expression of p-AKT was observed in the Wor+T4 and Per+T4 groups. No expression of p-mTOR and p-4E-BP1 was observed in the three groups, except for low expression levels in the Wor+T4 group at 0 h. No expression of p-p70S6K was observed in any of the groups, except for the control group. Very low expression levels of p-TSC2 were observed in the control group. **(c** and **d)** Similar changes were evident in A549/DDP cells.

Based on the above results, we further explored the effects of T4, wortmannin, perifosine and rapamycin on the PI3K/AKT/mTOR signaling pathway. The results showed that there were no differences among the Wor+T4, Per+T4, and Rap+T4 groups at 0 h and 24 h in the expression of PI3-K, PI3-P, AKT, TSC2, mTOR, p70S6K and 4E-BP1 in A549 lung cancer cells. However, the abundant expression of LC3 was detected when the cells were pretreated with wortmannin, perifosine or rapamycin and then treated with T4 for 24 h (Figure 4a). The same results were observed in A549/DDP lung cancer cells (Figure 4c).

In A549 lung cancer cells, a very low expression level of phosphorylated PI3-K (p-PI3-K) was observed in the Wor+T4 (at 0 h), Per+T4 (at 24 h), and Rap+T4 (at 24 h) groups; no expression of p-PI3-K was observed in the Wor+T4 group at 24 h; and the expression levels of p-PI3-K in the Per+T4 and Rap+T4 groups at 0 h were not significantly different than that of the control. A similar trend was observed in the results of p-PI3-P, which is a product of p-PI3-K in all groups except for the Wor+T4 group at 0 h. Regarding p-AKT, there were no significant differences between the Rap+T4 group and the control group; no expression of p-AKT was observed in the Wor+T4 and Per+T4 groups; no expression of p-mTOR and p-4E-BP1 were observed in the three groups, except for low levels of expression in the Wor+T4 group at 0 h; and no expression of p-p70S6K was observed in any of the groups, except for the control group. A very low expression level of p-TSC2 was observed in the control group; however, compared to the control, the expression of p-TSC2 was significantly increased in the Wor+T4, Per+T4, and Rap+T4 groups, particularly following a 24-h treatment of T4 (Figure 4b). A similar trend was observed in A549/DDP cells (Figure 4d).

**Figure 5:**
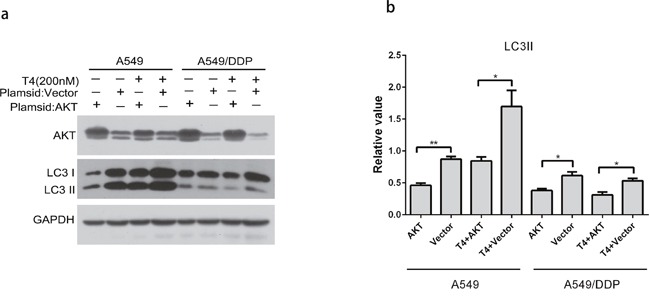
Changes in LC3 in A549 and A549/DDP cells following the OE-AKT+T4 treatment **(a** and **b)** LC3II increased when AKT was overexpressed, which was reversed by the T4 treatment (*: P<0.05, **: P<0.01).

AKT, which is also known as protein kinase B, acts as a nexus-signaling molecule in the receptor tyrosine kinase/phosphatidylinositol 3-kinase (RTK/PI3K) pathway and functions as an essential regulator of cell proliferation, survival, migration, and invasion [27]. To further verify our hypothesis, we adopted plasmid transfection to overexpress AKT in A549 and A549/DDP cells. Then, T4 was administered to the cells, and the protein was collected 24 hours later to detect the changes in LC3 expression by western blotting. The results showed that the expression of LC3II decreased when AKT was overexpressed and was upregulated when T4 was administered. Similar results were observed in A549/DDP cells (Figure 5a and 5b).

### Tripchlorolide effectively improves the sensitivity of A549/DDP cells to cisplatin

**Figure 6:**
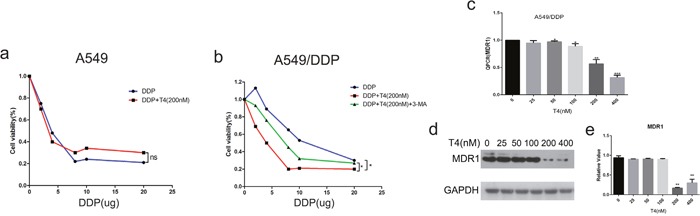
Effect of cisplatin on A549 and A549/DDP cells and the altered expression of MDR1 with increasing doses of T4 by Q-PCR and western blotting **(a** and **b)** Cisplatin induced more cell death in A549 cells. T4 accelerated cell death when the cells were pretreated with cisplatin, which was more noticeable in A549/DDP cells. **(c)** According to the Q-PCR analysis, T4 decreased the expression of MDR1 when its concentration reached 200 Nm (**: P<0.01, ***: P<0.001). **(d** and **e)** Western blotting revealed that T4 decreased MDR1 at the protein level (**: P<0.01).

The correlation between autophagy and drug resistance has been reported in the literature. As reported in the above-mentioned results, T4 had noticeable effects on cell autophagy. Thus, it is vital to examine the role of T4 in drug resistance reversal. Multidrug resistance has been a great impediment to successful cancer chemotherapy [28]. Currently, most lung cancer patients detected at an advanced stage are ineligible for surgical therapy. Chemotherapy is their first choice of treatment, but the therapeutic effect is not good. Drug resistance is a major obstacle to lung cancer treatment [29]. Therefore, efforts to improve the sensitivity of the patients to chemotherapy drugs are highly necessary. In our study, we measured the sensitivity of A549 and A549/DDP cells to cisplatin to ensure the drug-resistant action of A549/DDP for the subsequent experiments. In this analysis, we found that cisplatin killed A549 cells more effectively than A549/DDP cells (Figure 6a and 6b). Then, we assessed the effects of cisplatin on A549 cells and A549/DDP cells following the T4 treatment. Our results showed that the cell-killing effect of cisplatin on A549 cells did not increase after the T4 treatment, but this effect on A549/DDP significantly increased after the T4 treatment, and we found that the drug 3-MA could weaken the effect of T4 of improving the cisplatin sensitivity of A549/DDP Cells. (Figure 6a and 6b). These results illustrate that T4 can effectively improve the sensitivity of A549/DDP cells to cisplatin and proves that T4 improves cisplatin sensitivity in A549/DDP cells is by increasing the autophagy of A549/DDP Cells.

Because multi-drug resistance (MDR) is an essential aspect of human lung cancer chemotherapy failure [30], effectively reducing the expression of the MDR1 gene can increase patients’ sensitivity to chemotherapy drugs. Therefore, we detected the RNA expression levels of the MDR1 gene in A549/DDP cells after the T4 treatment. The result revealed that T4 decreased the expression of the MDR1 gene via QPCR (Figure 6c). To further confirm this finding, we examined the changes in the expression of the MDR1 gene at the protein level in A549/DDP cells. Our results showed that the expression of MDR1 decreased as the T4 concentration increased by western blotting (Figure 6d and 6e). The above-mentioned results confirm that tripchlorolide can effectively improve the sensitivity of A549/DDP cells to cisplatin, which may provide a good theoretical basis for treating lung cancer patients.

## DISCUSSION

T4 is a natural active compound extracted from tripterygium. We found that the T4 treatment significantly impaired the cell viability of A549 and A549/DDP lung cancer cells, and the cells that received the 3-MA pretreatment plus the subsequent T4 treatment revealed no significant differences in the cell viability, except for those exposed to 200 nM and 400 nM of T4 for 24-48 h. This finding indicates that T4 induced cell death mainly by autophagy, but there may be other mechanisms involved in the cell death of A549/DDP cells induced by T4 when the concentration of T4 was over 200 nM. We will explore other mechanisms in the future.

In this study, we explored the role of T4 in the autophagy of A549 and A549/DDP cells and the underlying mechanism. The results showed that T4 markedly reduced the activity of P13K and AKT, upregulated the expression of LC3II, and markedly affected the phosphorylated products of PI3-K, PI3-P, AKT, TSC2, mTOR, p70S6K and 4E-BP1 in A549 lung cancer cells, suggesting that T4-induced autophagy was closely correlated with the PI3K/AKT/mTOR signaling pathway and that T4 decreased the expression of MDR1 to improve the cisplatin sensitivity of A549/DDP cells.

Autophagy has attracted scientists' attention for decades. In the 1800s, an inverse correlation between malignant transformations was established. The autophagic activity of cancer cells was generally lower than the activity of normal cells. A decrease in autophagic activity can be induced by experimental carcinogenesis in animals or cultured cancer cell lines. The mechanism of the reduced autophagic capacity in cancer is complicated. In the 1990s, the PI3K/AKT/mTOR signaling pathway was considered the main pathway involved in the initiation and regulation of autophagy.

Class III PI3K is involved in the PI3K/AKT/mTOR signaling pathway, and its product is PI3P. PI3K activity has been reported to be important during the early stages of autophagic vesicle formation. The phenomena in which the inhibition of AKT induces autophagy and the activation of AKT inhibits autophagy have been observed in several studies. The tumor suppressor gene TSC2 is a negative regulator of autophagy [31]. TSC2 can inhibit mTOR signaling, which can be reversed by AKT phosphorylation. p70S6K and 4E-BP1 are downstream targets of mTOR; the inhibition and down-regulation of these molecules can induce autophagy.

To determine whether T4 induced autophagy in A549 and A549/DDP lung cancer cells through the inhibition of the PI3K/AKT/mTOR signaling pathway, cells were treated with T4 after pretreatment with PI3K, AKT or mTOR inhibitors (wortmannin, perifosine or rapamycin, respectively); the cell viability, PI3K and AKT activities, and expression of proteins involved in the PI3K/AKT/mTOR signaling pathway were measured. Our results suggest that T4 has a more obvious effect on the viability of A549 and A549/DDP lung cancer cells when combined with the rapamycin, wortmannin and perifosine pretreatment; PI3K is upstream of AKT and mTOR; and mTOR is downstream of PI3K and AKT in the PI3K/AKT/mTOR signaling pathway.

To better understand the T4-induced autophagy in A549 and A549/DDP lung cancer cells through the inhibition of the PI3K/AKT/mTOR signaling pathway, the effects of three inhibitors (wortmannin, perifosine and rapamycin for PI3K, AKT and mTOR, respectively) on the PI3K/AKT/mTOR signaling pathway were evaluated. In this study, autophagy was activated in A549 and A549/DDP lung cancer cells by wortmannin, perifosine and rapamycin and increased drastically when the cells were subsequently treated with T4 for 24 h, which is consistent with our previous results. The results also showed that all the phosphorylated products mentioned above in the PI3K/AKT/mTOR signaling pathway were affected by wortmannin; only PI3-K and PI3-P were affected by perifosine; and only p70S6K and 4E-BP1 were affected by rapamycin, demonstrating that PI3K is upstream of AKT and mTOR and that mTOR is downstream of AKT in the PI3K/AKT/mTOR signaling pathway. Additionally, we adopted AKT plasmid transfection in A549 and A549/DDP lung cancer cells to overexpress AKT, which decreased autophagy. When T4 was administered to the cells, LC3 expression was upregulated, and autophagy was increased.

In the current study, we showed that autophagy in A549 and A549/DDP lung cancer cells could be induced by T4, which had a better effect on autophagic activities with PI3K, AKT and mTOR inhibitors, and the T4-induced autophagy was facilitated through the inhibition of the PI3K/AKT/mTOR signaling pathway. Because the mechanism underlying this inhibition is complex, we hypothesize that T4 induces autophagy by inhibiting PI3-K phosphorylation and its product p-PI3-P, the phosphorylation of AKT. The inhibition of p-AKT, in turn, increases the expression of p-TSC2 and down-regulates p-mTOR, which is followed by the down-regulation of phosphorylated p70S6K and 4E-BP1, to induce autophagy. Resistance to chemotherapy drugs is the main reason for failed chemotherapy. MDR1 is considered an extremely important molecular target for drug resistance in cancer therapy [28]. In our study, we found that cisplatin had a better killing effect on A549/DDP cells after the T4 treatment and that T4 decreased the RNA and protein expression of MDR1. Therefore, we claim that T4 can improve cisplatin sensitivity in A549/DDP cells. It is known that PI3K/AKT is the main downstream signaling pathway in patients with EGFR mutations. Currently, there is a consensus among clinicians that epidermal growth factor receptor (EGFR) tyrosine kinase inhibitors (TKIs) have a favorable efficacy in NSCLC patients with EGFR mutations, and some relevant research studies have suggested that the presence of EGFR mutations is a favorable prognostic marker [32]. However, the association between the EGFR mutation status and the responsiveness to conventional chemotherapy agents and survival in NSCLC patients remains unclear. Therefore, it will be interesting to obtain additional data regarding the effect of tripchlorolide in a cell line with EGFR mutations or ALK fusion in our future research. Moreover, because of the close correlation between cancers and reduced autophagic activities, further studies that explore the possibility of developing new therapeutic methods for cancers and resistant diseases are eagerly anticipated.

Altogether, the current study reports for the first time that T4 can induce autophagy in A549 and A549/DDP cells via the inhibition of the PI3K/AKT/mTOR signaling pathway and can increase cisplatin sensitivity in A549/DDP cells. In the future, we will strengthen our animal and clinical experiments, which may provide theoretic evidence for the development of effective clinical therapeutic treatments for cancers and resistant diseases.

## MATERIALS AND METHODS

### Materials and reagents

Tripchlorolide was purchased from Amresco (Amresco, CA, USA). The cell-counting Kit-8 (CCK-8) assay was purchased from Dojin- do (Dojin- do, Japan), and dimethyl sulfoxide (DMSO) was purchased from Sigma (St. Louis, MO, USA). Wortmannin (a PI3K inhibitor), perifosine (an AKT inhibitor) and rapamycin (a mTOR inhibitor) were obtained from Selleck (Selleck Chemicals, CA, USA). Antibodies against PI3K, PI3P, AKT, TSC2, P70S6K, and 4E-BP1 and their corresponding secondary antibodies were obtained from Santa Cruz (Santa Cruz, CA, USA). An enhanced chemiluminescence (ECL) kit was purchased from PerkinElmer (Waltham, MA, USA). The AKT activity was measured using a KinaseSTARTM AKT activity assay kit (BioVision). A PI3K enzyme-linked immunosorbent assay kit was obtained from Echelon Bioscience.

### Cell culture

A549 and A549/DDP lung cancer cells (cisplatin-resistant lung cancer cell lines) were obtained from the Cell Line Bank, Chinese Academy of Sciences. A549 cells were cultured in DMEM- high glucose supplemented with 10% fetal bovine serum (FBS) and 100 μg/mL penicillin/streptomycin in a humidified incubator under 5% CO2 at 37°C. A549/DDP cells were cultured in RPMI-1640 supplemented with 10% fetal bovine serum (FBS) and 100 μg/mL penicillin/streptomycin in a humidified incubator under 5% CO2 at 37°C. The cell culture media were replaced with fresh media every two days.

### Drug treatments

A549 and A549/DDP lung cancer cells were seeded in 6-well plates at a density of 4×105 cells per dish and randomly divided into the following five groups: i) the control group, with no drug treatment; ii) T4 group, exposed to T4 at a final concentration of 200 nM; and iii-v) Wor+T4 group, Per+T4 group, and Rap+T4 group, which received wortmannin, perifosine or rapamycin pretreatment plus a subsequent T4 treatment, respectively, in which the cells were pretreated with a series of concentrations of wortmannin, perifosine or rapamycin (0-800 nM) for 1 h and subsequently treated with T4 (200 nM). The cells were collected after they were treated with/without T4 for 24 h. Each experiment was performed in triplicate.

### CCK8 assay

The cells were seeded onto 96-well plates at a density of 5×103 and were incubated for 24 h. The cells were then exposed to wortmannin, perifosine or rapamycin at various concentrations; After one hour, T4 was added to each well. After twenty-four hours, CCK8 was added to each well, and the plate was re-incubated at 37°C for 1-4 h. The absorbance value was analyzed at 595 nm with a microplate reader. The cell viability rate was calculated according to the following formula: viability rate=A595(experimental group)/A595(control group)×100%. Viability rate=A595/DDP(experimental group)/A595/DDP(control group)×100%. Each experiment was performed in triplicate.

### Western blotting

The cells were collected and lysed in a lysis buffer (pH=7.4, containing 0.1% SDS, 100 mM NaCl, 1% Triton-X 100, 10 mM Tris, 1 mM NaF, 2 mM Na3VO4, 20 mM Na4P2O7, 1 mM EGTA, 1 mM EDTA, 0.5% sodium deoxycholate, 1 mM PMSF, 60 μg/mL aprotinin, 10 μg/mL leupeptin, and 1 μg/mL pepstatin) on ice for 30 min before centrifugation and quantification. The supernatants were separated using SDS-PAGE (10%) gel electrophoresis and transferred onto a PVDF membrane. The membrane was subsequently incubated with 5% non-fat milk in TBS for 1 h to block the nonspecific binding sites and then incubated with the primary antibody (diluted 1:1000) overnight at 4°C and then an appropriate peroxidase-conjugated secondary antibody (coat anti-rabbit HRPO, diluted 1:2000) for 1 h. After the final washing, the signal was developed using an ECL detection system. The images of the bands were quantified and assessed with the software Image J. Each experiment was performed in triplicate.

### PI3K activity assay

PI3K activity was measured using a PI3K enzyme-linked immunosorbent assay kit according to the manufacturer's instructions. PI3K can convert PI(4,5)P2 to PI(3,4,5)P3. The activity of PI3K was measured using a thin layer chromatography (TLC)-based assay that used PI(4,5)P2 as a substrate. The cells were rinsed with an ice-cold buffer and harvested with an ice-cold lysis buffer. Then, PI3K was isolated from the cellular proteins by immunoprecipitation using a PI3K antibody. The immunocomplex-bound protein A-agarose beads were incubated in a reaction buffer containing PI(4,5)P2 and ATP, and the kinase reaction was stopped by centrifugation. The absorbance value of the reaction mixtures was measured at 450 nm with a microplate reader. The PI3K activity was calculated from a standard curve using various concentrations of PI(3,4,5)P3.

### AKT activity assay

The cell lysate was prepared using a kinase extraction buffer. For each assay, 2 μL of an AKT-specific antibody were added to 200 μL of the cell lysate, and the mixture was rotated at room temperature for 45 min. Then, 50 μL of a protein A sepharose slurry were added to each sample, and the samples were rotated at room temperature for 1 h. The protein A beads were obtained by centrifugation and washed with a kinase extraction buffer and a kinase assay buffer. Then, 2 μL of the GSK-3α protein/ATP mixture were added, and the mixture was incubated at 30°C for 1-4 h. The protein A beads were spun, and 30 μL of the supernatants were collected in a new Eppendorf tube. Then, 15 μL of 3×SDS-PAGE buffer were added. The samples were boiled for 3 min, and the protein A beads were again spun. The supernatant (20 μL) was loaded onto a 12% SDS-PAGE gel. Alternatively, the supernatant was stored at −20°C for future use. Western blotting was performed using the rabbit anti-phospho-GSK-3α (Ser 21)-specific antibody at a 1:1000 dilution. A 37-kDa band corresponding to phosphorylated GSK-3α was detected in the AKT-activated samples.

### Quantitative real-time PCR

A549 cells and A549/DDP cells were subjected to total RNA extraction using a commercially available assay (TriPure Isolation Reagent, Roche) according to the manufacturer's protocol. First-strand cDNA was synthesized using 1 μg of total RNA (Transcriptor First Strand cDNA synthesis kit, Roche). The quantitative PCR was performed on a Step One Applied Biosystems (Applied Biosystems, Foster City, CA) using SYBR Green Master (Roche) to measure the fluorescence intensity of the amplified products. The reactions were performed as follows: 55°C for 2 min, 95°C for 10 minutes, and then 40 cycles of 95°C for 15 seconds, followed by 60°C for 1 minute. The data were analyzed by the ΔΔCt method, and gapdh served as a housekeeping gene.

### AKT over-expression

Cells were incubated overnight to achieve 70% confluency before the siRNA transfection. After the transfection, the cells were incubated with the transfection mixture for 24 h. The cells were incubated for 48 h before harvesting.

### Statistical analysis

The data were expressed as the mean±SD (standard deviation) of triplicates. Statistical significance of the differences throughout this study was assessed by one-way ANOVA. All analyses were conducted using SPSS 13.0 software. P-values<0.05 were considered statistically significant.

## Abbreviations

T4: tripchlorolide
PI3K: phosphatidylinositol 3-kinase
AKT: protein kinases B
A549/DDP: A549 cisplatin-resistant cell lines
Wor: wortmannin,
Per: perifosine
Rap: rapamycin
3-MA: 3-Methyladenine
NSCLC: non-small-cell lung cancer
ATP: adenosine-triphosphate
TEM: transmission electron microscope
MDR1: multidrug resistance protein 1.
